# Diphtheria and Bacteria

**Published:** 1882-03-20

**Authors:** 


### DIPHTHERIA AND BACTERIA.

---

The supplement to the National Board of Health Bulletin for January 21st, contains the full report of the experiments conducted by Drs. H. C. Wood and H. F. Formad, of Philadelphia, under the auspices of the Board, with a view of determining the connection of diphtheria with fungi. This report is one which is a credit to American medicine, and would place Drs. Wood and Formad in a high place among the careful observers in any country. It is an eminently scientific report, and although the experiments undertaken have not yet been as conclusive in their results as one could wish, there is that in the record of them which inspires faith in the ultimate practical good whieh will cro ·yn the labors of these observers.

The experiments were undertaken to determine two questions: First, whether bacteria, which are always present in the false membrane of diphtheria, are identical in form and size with those which are present, not only in the membrane of non-diphtheritic tracheitis, but also in the inflammation of an inflamed tonsil; second, whether bacteria are always present in diphtheria, or only in some cases. To answer the first question a large number of cases were examined. These cases are divided into two sets: First, those of endemic or sporadic diphtheria; second, those of true malignant epidemic diphtheria. The experiments consisted of two sets, the first being conducted on eight mild cases occurring in Philadelphia, and the second on fourteen cases of malignant diphtheria as it occurred in epidemic form at Ludington in this State. Out of the seven cases, in which blood was examined during life for micrococci, in six no fungi was found, while in one case micrcocci were somewhat abundant in the vital fluid. Of the Ludington cases, fourteen in number, in seven micrococci were found, and in seven none were present. The cases in which there were no micrococci were all of them very light, or in the stage of convalescence, and the amount of fungi present in the malignant cases seemed to be proportionate to the severity of the symptoms, and to steadily progress with the disease in the fatal cases. "The study of these two sets of cases is sufficient," they say, "to enable us to formulate, as established, the proposition that in endemic mild diphtheria, micrococci are always present in the part locally diseased, but are usually not present during life in the blood, or in the glandular organs, even in cases which prove fatal; that in malignant epidemic diphtheria micrococci are always present in the part locally diseased, and are also usually, and perhaps always to be found in the blood and tissues of severe cases, but are frequently, if not usually, absent from the blood of mild cases."

Observations conducted to determine whether micrococci are fonnd in the blood of other diseases than diphtheria, and if so whether they are distinguishable from those found in the latter disease, were not conclusive.

Their testimonv on that much mooted question, the identity of diphtheria and croup, is far from fortifying the position assumed by the dualist.

They say "It will be shown that the morbid cases which give rise in the respective lesions in the pharynx and in the air passages is the same, and the anatomy of the products identical." They claim that the apparent difference in the lesions and in the morphology of the exudates is altogether conditioned by and dependent upon the anatomical peculiarities of the pharynx and respiratory passage. The reasons which they give for such an expression are fully stated and are indeed very conclusive as arguments in favor of the identity of the two diseases.

The following summary is given of the chapter on the "Nature of Diphtheria:"

"The micrococci of diphtheria do not differ, so far as observed, from the micrococci of furred tongue, etc., except in their tendency to grow in culture fluids.

The micrococci of furred tongue or ordinary sore throat have a less tendency to grow under culture than have the micrococci of endemic non-malignant diphtheria.

The micrococci of endemic or non-malignant diphtheria have a much less tendency to grow under culture than have the micrococci of malignant diphtheria.

The rapidity of growth of the micrococci is in direct proportion to the malignancy of the cases yielding them, and its contagiousness.

On exposure to the air diphtheritic members of the most violent type loses its contagious power, and the micrococci *pari passu* lose their power of growing in culture fluids.

Under successive generations of artificial culture the diphtheritic micrococci lose their growth, activity and also their power of infecting the rabbit.

It has not been experimentally directly proven, but it is a necessary inference from the two facts just stated, that under certain favoring circumstances the sluggish micrococci puts on growth, activity, and, in all probability, *poisonous properties.*

Every grade of case can be found in man from an ordinary sore throat, through simple pseudo-membranous angina and tracheitis up to malignant diphtheria.

Any inflammation of the trachea of sufficient intensity may cause the formation of pseudo-membrane."

If these experiments teach anything, it is that diphtheria is not a specific disease, or rather that the micrococcus which has been regarded as essential to the existence of the disease is not of a different nature from that found in healthy throats, but that in diphtheria this micrococcus has been stirred up into unwonted activity, the soil furnished by individual throats favoring such activity, and the rapid growth of the germ. They teach that the micrococcus finds a favorable soil in throats which would naturally be supposed to have the least powers of resistance, *e. g.*, in children and in debilitated adults. They teach also that diphtheria is first a local disease before it is a systemic, and inculcate the importance of early local treatment with a view to destroying the bacteria before they find an entrance into the circulation and to cause systemic infection. There is, of course, nothing new in these deductions, but the experiments give them increased weight and will lead to their more general acceptance.—*Michigan Medical News.*

